# From Nature to Pharmacy: A Review of Tectoridin for Modern Therapeutics

**DOI:** 10.3390/ph19050703

**Published:** 2026-04-29

**Authors:** Shengxi Zhang, Jinxi Huang, Xiaoming Li, Ziling Zhou, Shichang Bai, Dan Zhang, Tao Song, Xianyao Wang, Jun Tan, Qinghong Kong, Jidong Zhang, Changxin Li

**Affiliations:** 1 Department of Immunology, Zunyi Medical University, Zunyi 563000, China; zhangshengxi219@gmail.com (S.Z.); huangjinxi2024718@126.com (J.H.); lixm310predoom@163.com (X.L.); zhouziling1115@163.com (Z.Z.); 18341413828@163.com (S.B.); songtao@zmu.edu.cn (T.S.); wangxianyao@zmu.edu.cn (X.W.); 2 Zunyi Medical University Library, Zunyi 563000, China; tsgbgs@zmu.edu.cn; 3 Department of Histology and Embryology, Zunyi Medical University, Zunyi 563000, China; juntan_zmu@126.com; 4 Guizhou Provincial College Based Key Lab for Tumor Prevention and Treatment with Distinctive Medicines, Zunyi Medical University, Zunyi 563000, China; kqinghong2023@126.com; 5 Collaborative Innovation Center of Tissue Damage Repair and Regeneration Medicine, Zunyi 563000, China; 6 Department of Rehabilitation Medicine, Affiliated Hospital of Zunyi Medical University, Zunyi Medical University, Zunyi 563000, China

**Keywords:** tectoridin, pharmacology, toxicity, cancer, inflammatory diseases

## Abstract

**Background:** Tectoridin is a prominent isoflavone glycoside found in herbs such as *Belamcanda chinensis* (L.) DC and Iris tectorum Maxim. It has drawn increasing research interest due to its promising pharmacological activities. However, no critical review to date has determined whether its broad pharmacological activity stems from binding to specific targets or from the non-specific, broad-spectrum activity commonly associated with flavonoids. This paper provides a comprehensive review of tectoridin, covering its plant sources, pharmacological effects, pharmacokinetics, and toxicity, alongside an in-depth analysis of the mechanisms underlying its pharmacological effects and strategic recommendations for advancing its clinical translation. **Methods:** A systematic literature search was conducted in PubMed, Web of Science, Google Scholar, SciFinder, and CNKI for publications from 1968 to 2025 using keywords including tectoridin, tectorigenin 7-O-glucoside, traditional uses, ethnopharmacology, pharmacology, bioactive compounds, biological activity, pharmacokinetics and toxicity. **Results:** Tectoridin exhibits a broad spectrum of pharmacological activities, including anticancer, anti-inflammatory, hepatoprotective, antidiabetic, antioxidant, cardiovascular, and estrogenic effects. Pharmacokinetic studies have shown rapid tissue distribution and slow elimination; the aglycone metabolite tectorigenin often displays enhanced bioactivity, and chemical modifications may further improve efficacy. Toxicity data suggest relative safety in medicinal food contexts, but comprehensive in vivo studies remain limited. Tectoridin shows promise for treating cancer and inflammatory diseases; however, further research is needed to elucidate its molecular mechanisms, clarify toxicity, and optimize bioactivity. **Conclusions:** This review bridges natural products and modern therapeutics by focusing on tectoridin, highlighting its therapeutic potential, addressing challenges, and offering new perspectives for treating various diseases.

## 1. Introduction

**Figure 1:**
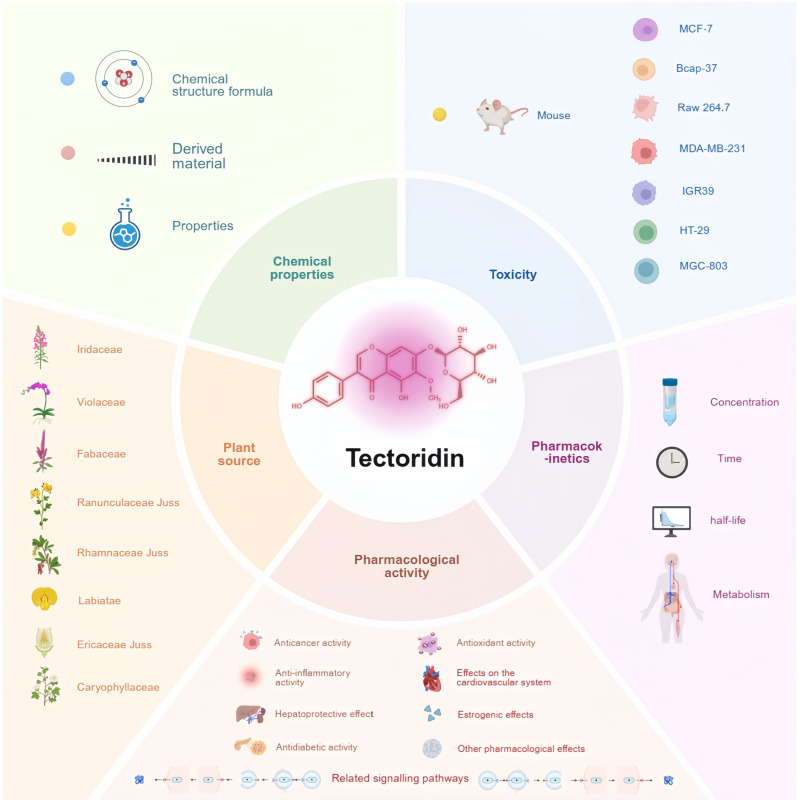
Overview diagram of tectoridin. The diagram is divided into five sections describing the chemical properties, plant origin, biological activity, pharmacokinetic profile, and toxicity of tectoridin.

Plant-derived natural products remain a vital source for developing treatments for various diseases, and recent research increasingly demonstrates their contribution to innovative therapeutic development [1]. Isoflavones are polyphenolic non-steroidal estrogenic compounds widely found in plants and have attracted significant attention due to their important biological activities [2]. Tectoridin is a natural isoflavone glycoside present in many Chinese herbal medicines, such as *Belamcanda chinensis* (L.) DC and *Iris tectorum Maxim* [3,4]. Recent studies have extensively investigated the pharmacological activities of tectoridin, including anticancer, anti-inflammatory, hepatoprotective, antidiabetic, antioxidant, cardiovascular, and estrogenic effects. These findings suggest that tectoridin could serve as a potential therapeutic agent for conditions such as cancer, inflammatory diseases, alcoholic liver disease, diabetes, and cardiovascular diseases. Although the pharmacological effects of tectoridin are well-documented, existing reports remain scattered and unsystematic. This review integrates current knowledge on its pharmacological activities, pharmacokinetics, and toxicity to inform future development and clinical translation. An overview is provided in Figure 1.

Furthermore, beyond providing a comprehensive review, this paper identifies several unresolved and critical issues in the field; these issues not only hinder our understanding of tectoridin’s mechanism of action but also obscure the research direction for its translation from nature to the pharmaceutical industry. For example, according to previous reports, tectoridin modulates a wide range of signalling pathways and exhibits various effects, including anti-inflammatory, anti-cancer, antioxidant, hepatoprotective and oestrogen-like activities. However, these characteristics are typical of many polyphenolic flavonoids; this multifunctional pharmacological activity may not stem from discrete, high-affinity binding to specific protein targets, but rather from non-specific physicochemical properties. Consequently, this paper provides a critical evaluation of tectoridin’s specific pharmacological activity to determine whether the effects observed with tectorigenin reflect genuine, targeted regulation of specific signalling nodes, or are primarily attributable to the non-specific, multi-pharmacological behaviour inherent to the flavonoid chemical class. The aim is to distinguish the true pharmacological mechanisms of tectoridin from the background biological effects common to the entire class of flavonoids, thereby providing new theoretical guidance for the clinical translation and development of tectoridin.

## 2. Materials and Methods

### 2.1. Search Strategy

A systematic literature search was performed across multiple electronic databases, including PubMed, Web of Science, and Embase, covering the period from database inception to 13 October 2025. The search strategy combined terms for the compound with terms for pharmacological outcomes using Boolean operators. The primary search string was: (“tectoridin” OR “tectorigenin 7-O-glucoside”) AND (“pharmacology” OR “pharmacokinetics” OR “toxicity” OR “traditional uses” OR “ethnopharmacology” OR “bioactive compounds” OR “biological activity”). No restrictions were applied regarding date, language, or study design. No published search filters were used. During the information search, no attempts were made to contact authors, experts, manufacturers or other relevant parties to seek additional research or data.

### 2.2. Inclusion and Exclusion Criteria

Studies were included if they met the following criteria: (1) original research exploring the pharmacological effects of tectoridin; (2) studies conducted in vivo (animal models) or in vitro (cell lines); and (3) articles providing clear data on molecular mechanisms or therapeutic outcomes. Exclusion criteria were: (1) case reports and conference abstracts; (2) studies using compound formulas where Tectoridin was not the primary focus; and (3) duplicate publications or studies with inaccessible full texts.

### 2.3. Selection and Data Collection

Two reviewers independently screened the titles and abstracts of the retrieved records. Potentially relevant studies then underwent full-text assessment based on the pre-defined inclusion criteria. Any discrepancies between the reviewers were resolved through discussion or consultation with a third senior reviewer. Data from the included studies were extracted using a standardized form, including information on the author, year, model/cell type, dosage, intervention, and key signaling pathways (e.g., MAPK, NF-κB).

### 2.4. Prior Work and Updates

This search strategy was developed de novo for this specific review and was not adapted or reused from a prior literature review. No formal email alerts or auto-updates were established after the final search date.

### 2.5. Peer Review of Search Strategy

The search strategy was peer-reviewed by a second information specialist (J.D.Z.) before execution using the PRESS (Peer Review of Electronic Search Strategies) Checklist to ensure appropriate use of Boolean operators, syntax, and subject headings.

### 2.6. Managing Records and Study Selection

A total of 206 articles were identified through the literature search. After removing duplicate entries and adjusting the inclusion criteria, excluding studies with outdated methodologies, excessively long durations, or duplicate content, 111 articles were ultimately determined to meet the inclusion criteria for this review.

Following automated deduplication, a manual review of the remaining records was conducted by two reviewers (S.Z. and J.H.) to identify and merge any residual duplicate entries missed by the algorithm (e.g., variations in journal name abbreviations).

## 3. Chemical Properties and Phytogenic Origin of Tectoridin

**Figure 2:**
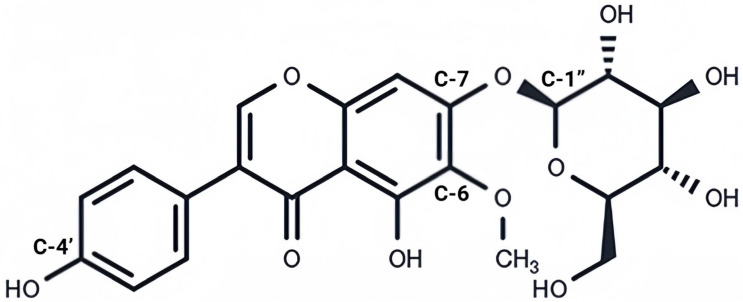
Chemical structures of tectoridin. Presentation of the complete chemical formula of tectoridin.

Tectoridin is a well-known isoflavone glycoside with the molecular formula C_22_H_22_O_11_, identified as a 4′,5,7-trihydroxy-6-methoxyisoflavone-7-O-β-D-glucopyranoside [5]. Several structurally similar derivatives exist, including tectorigenin, 6′-O-xylosyl-tectoridin, tectoridin A, and tectorigenin 7-O-β-D-glucopyranosyl-12-O-β-D-glucopyranoside [3,6,7]. Its chemical structure is shown in Figure 2.

Tectorigenin, the aglycone of tectoridin, is formed by removal of the glucosyl group at the C-7 position [8]. Tectoridin can be further glycosylated at the 6′ position of the glucose moiety to form 6′-O-xylosyltectoridin [3,6,7]. Tectoridin is sparingly soluble in water, and its solubility decreases when converted to tectorigenin. However, introducing a sodium sulfonation moiety into tectorigenin significantly enhances water solubility [5].

Tectoridin occurs in several plants, primarily the rhizome of *Belamcanda chinensis* (L.) DC [9,10,11] and Iris tectorum Maxim [10,12,13,14]. Many other Iris species are also major sources [8,15,16,17,18,19,20,21,22,23]. Tectoridin is also found in plants from other families, such as *Viola mandshurica* (Violaceae) [24], *Pueraria species* (Fabaceae) [6,7,25,26,27,28,29], *Astragalus membranaceus* (Fabaceae) [29,30], and others [31,32,33,34,35,36]. The plant sources of tectoridin are summarized in Table 1.

**Figure 3:**
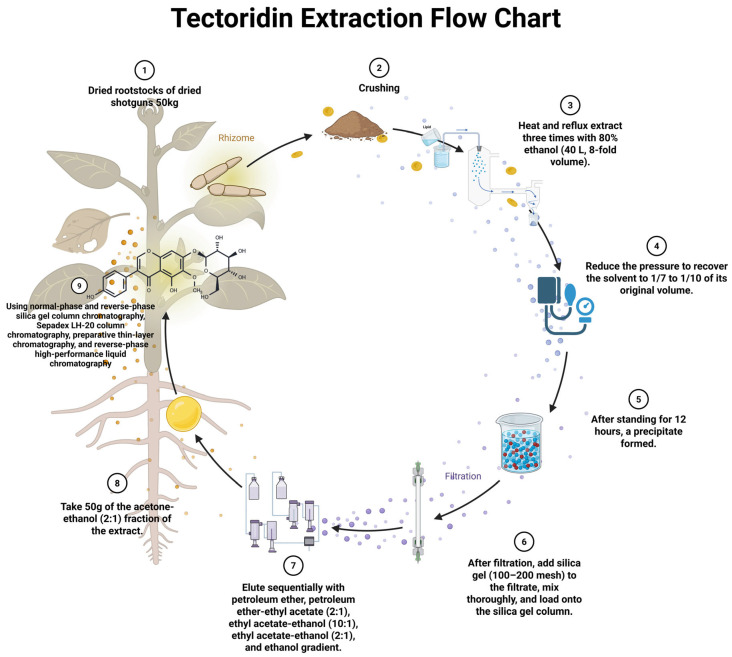
Flowchart for the isolation of tectoridin from *Belamcanda chinensis*. ① Take 50 kg of dried rhizome of *Belamcanda chinensis*. ② Pulverise. ③ Extract with 8 times volume, i.e., 40 L of 80% ethanol by heating and refluxing for 3 times. ④ Recover the solvent under reduced pressure to 1/7~1/10 of the original volume. ⑤ Leave it for 12 h until precipitation occurs. ⑥ After filtration, the filtrate was added with silica gel (100–200 mesh) and the sample was stirred on a silica gel column. ⑦ Eluted with petroleum ether, petroleum ether-ethyl acetate (2:1), ethyl acetate-ethanol (10:1), ethyl acetate-ethanol (2:1), and ethanol gradient, respectively. ⑧ Take 50 g of ethyl acetate-ethanol (2:1) part of the extract. ⑨ Using normal and reversed-phase silica gel column chromatography, SephadexLH-20 column chromatography, preparative thin-layer chromatography, and reversed-phase HPLC, the separation of tectoridin was obtained.

General steps for isolating tectoridin from plants, using *Belamcanda chinensis* as an example, are shown in Figure 3.

## 4. Pharmacological Activities of Tectoridin

### 4.1. Anticancer Activity

Cancer remains a leading cause of mortality worldwide. Many studies indicate that tectoridin exerts anticancer effects through various mechanisms. Xiong et al. [37] found that tectoridin dose-dependently inhibited colon cancer cell proliferation by modulating apoptosis-related proteins, decreasing Bcl-2 and p53 expression, and increasing Bax expression. Tectoridin also inhibited the PKC/p38MAPK pathway, reducing colon cancer progression [37]. Wang et al. found that tectoridin can synergize with PLK1 inhibitors to promote apoptosis in lung adenocarcinoma cells [38]. Additionally, 10 µM tectoridin significantly inhibited RM-1 cell growth by 60%, and 100 µM tectoridin inhibited H22 cell growth by 12% [39], suggesting potential anti-prostate cancer activity that warrants further investigation.

The PI3K/AKT pathway plays a pivotal role in tumorigenesis [40]. Zhang et al. found that tectoridin suppressed bladder cancer cell viability dose-dependently by targeting RAB27B to regulate the PI3K/MAPK pathway. Wang et al. demonstrated that tectoridin inhibited proliferation, migration, and invasion of ovarian cancer SK-OV-3 cells by modulating the PI3K/AKT signaling pathway [41]. Collectively, These findings indicate that tectoridin holds significant potential in cancer therapy, and its effects on core pathways such as PI3K/AKT and MAPK may offer novel strategies for addressing cancer treatment challenges.

It is worth noting, however, that whilst the aforementioned studies confirm that tectoridin has considerable potential in cancer treatment, the translational significance of these findings is severely limited by pharmacokinetic constraints. In vitro, tectoridin and tectorigenin typically inhibit cell viability, induce apoptosis and impair migration at concentrations ranging from 10 to 100 µM [36,37,38,39,40,41]. Studies have shown that after oral administration of tectoridin (200 mg/kg) to rats, the parent compound reaches a peak plasma concentration of approximately 50 µM only briefly, with the major circulating forms being the glucuronide and sulphate conjugates of tectorigenin [42,43]. Consequently, achieving in vivo concentrations that exhibit direct cytotoxicity equivalent to those observed in vitro is difficult; any in vivo anticancer efficacy is more likely to stem from indirect mechanisms such as gut modulation or immune modulation. Furthermore, there is inconsistency in the existing literature: some studies provide detailed pathway analysis and genetic validation (such as RAB27B knockdown in bladder cancer [40]), whilst others merely report growth inhibition at the phenotypic level without exploring the underlying mechanisms [39]. Crucially, no studies have yet evaluated the efficacy of oral tectoridin in tumour models of the same or different species, nor have any studies systematically compared the activity of tectoridin and tectorigenin in multiple cancer cell lines under standardized conditions. Thus, the anticancer potential of tectoridin still requires rigorous in vivo validation.

### 4.2. Anti-Inflammatory Activity

Inflammation is implicated in numerous diseases, such as pneumonia and severe pancreatitis. Tectoridin exhibits significant anti-inflammatory activity. Zhou et al. examined its effects in LPS (Lipopolysaccharide, LPS)-induced severe acute pancreatitis and found that tectoridin reduced pancreatic injury, lowered serum amylase and lipase levels, inhibited macrophage M1 polarization, and decreased TNF-α levels [44]. Niu et al. [13] reported that tectoridin alleviated tissue injury in LPS-induced endotoxic shock by lowering IL-6 and IL-1β levels, demonstrating a protective effect similar to dexamethasone. This effect was mediated by suppression of the TLR4-NF-κB/NLRP3 axis [13]. Furthermore, tectoridin attenuated TNF-α-induced hyper-proliferation and inflammatory responses in rheumatoid arthritis fibroblast-like synoviocytes by inhibiting the TLR4/NLRP3/NF-κB pathway [45]. Huang et al. showed that tectoridin reduced paw and joint swelling, attenuated tissue damage, and inhibited pro-inflammatory cytokine secretion in rheumatoid arthritis mice by targeting ERK, JNK, and p38 phosphorylation [4]. Phospholipase A2 (PLA2) catalyzes the hydrolysis of membrane phospholipids, and its inhibitors are potential therapeutic agents for inflammation-related disorders. Shikimic acid glycosides interact with PLA2 active sites, confirming their anti-inflammatory efficacy [46].

Many tectoridin-containing herbs are used to treat inflammatory diseases. *Belamcanda chinensis*, a primary source of tectoridin, is used against asthma and exhibits anti-inflammatory and immunomodulatory properties [47]. The Flos Puerariae-Semen Hoveniae medicine pair, which contains tectoridin, improves inflammatory bowel disease by regulating inflammatory cell infiltration and cytokine secretion [48]. Gao and Ma et al. found that tectoridin showed 69.7% NO inhibitory activity in an LPS-induced RAW264.7 macrophage model [49]. Liang et al. identified tectoridin as the core ingredient of Ganke Granules, which inhibit LPS-induced inflammatory response in acute lung injury via the SRC/ERK1/2/STAT3 pathway [50]. Kim et al. demonstrated that fermented Viola mandshurica extract, rich in tectoridin, suppressed NO generation, iNOS expression, PGE2 production, COX-2 expression, and ERK and JNK pathway activation [24].

Taking the above research findings into account, tectoridin exhibits significant anti-inflammatory activity and shows potential for the treatment of inflammation-related diseases. It is worth noting that the activity of tectorigenin is superior to that of the parent glycoside in various experimental systems—for example, in rat peritoneal macrophages, tectorigenin exhibits a stronger inhibitory effect on prostaglandin E2 production by suppressing COX-2 induction than tectoridin [51], further highlighting the functional importance of deglycosylation. In contrast to cancer research, oral administration of tectoridin does indeed produce significant anti-inflammatory effects in vivo; this is most likely because the target tissues are precisely where metabolite concentrations are highest following oral administration. However, a fundamental ambiguity remains unresolved: it is not yet conclusively established whether the observed pathway inhibition reflects the direct binding of tectoridin to upstream kinases or is secondary to an antioxidant response. The concentrations required for in vitro anti-inflammatory activity (typically 10–50 µM) remain higher than the steady-state systemic concentrations of free tectorigenin, suggesting that local tissue accumulation or an amplification of redox signalling sensitivity may explain the manifestation of in vivo efficacy. Future research is needed to further distinguish between direct target binding and pathway regulation.

### 4.3. Hepatoprotective Effect

The liver is central to bile production, metabolism, and toxin degradation. Traditional Chinese medicine (TCM) shows promise in treating liver diseases due to its efficacy and low side effects [52]. Alcoholic liver disease (ALD) is a common clinical concern [26]. Puerariae Flos, used clinically for ALD [27], contains tectoridin as a key hepatoprotective component [7]. The hepatoprotective effects involve microbial transformation of tectoridin and bioactivation of its metabolites [53].

Xiong et al. found that tectoridin attenuated intracellular fat accumulation in hepatocytes, reduced AST (Aspartate aminotransferase, AST) and TG (Triglyceride, TG) levels, improved mitochondrial dysfunction, suppressed TBARS (Thiobarbituric acid reactive substances, TBARS) overproduction, and reversed downregulation of PPARα and its target genes [54]. Nayeema Akther et al. identified tectoridin as the major compound in *Iris* spp. extracts, which restored hepatic tissue structure at doses of 100 and 200 mg/kg [55]. Zhou et al. reported that *Iris tectorum* Maxim. exhibited stronger hepatoprotective activity than *Belamcanda chinensis*, likely due to higher tectoridin content [10]. Oral tectoridin inhibited β-glucuronidase and CCl_4_-induced hepatotoxicity in mice [56]. Lee et al. also showed hepatoprotective effects of orally administered tectoridin in t-BHP-injured HepG2 cells and mice [57].

These findings not only highlight tectoridin’s hepatoprotective potential but also suggest that tectoridin acts as a multi-target modulator, exerting effects on the core pathological processes of liver disease: steatosis, oxidative stress, and inflammation. Furthermore, tectoridin shows great promise for translation in terms of its hepatoprotective effects, having demonstrated consistent in vivo efficacy across multiple models of liver injury; crucially, the hepatoprotective effect of orally administered tectoridin is lost when given via intraperitoneal injection, directly demonstrating that its deglycosylation by the gut microbiota to form tectorigenin is an essential activation step; furthermore, following oral administration, tectoridin is able to provide effective hepatoprotection despite its extremely low systemic bioavailability [57]. At the cellular level, tectoridin reduces lipid accumulation in hepatocytes, decreases the release of AST and ALT, restores PPARα expression and improves mitochondrial function [54]. These multi-target effects likely reflect a synergistic interaction between direct metabolic regulation and Nrf2-mediated antioxidant defence, rather than binding to a single molecular target. However, there is currently a lack of research using clinically relevant models of chronic liver disease, as well as a lack of dose–response characterisation in large animals. Furthermore, whilst the pharmacokinetic advantages of portal vein delivery are significant, they also present a limitation: without a targeted delivery strategy, tectoridin is unlikely to provide meaningful protection to extrahepatic organs. Future development should prioritize formulation strategies that leverage the enterohepatic circulation.

### 4.4. Antidiabetic Activity

In China, several herbs containing tectoridin (e.g., *Astragalus membranaceus*, *Belamcanda chinensis*, *Pueraria lobata*) are used to treat diabetes. Liu et al. identified flavonoids, including tectoridin, as the main active components of Astragalus [30]. Chen and Wu et al. demonstrated hypoglycemic and anti-hyperglycemic activity of *Belamcanda chinensis* leaf extract in normal and diabetic rats [58], with tectoridin identified as a principal active flavonoid [59]. *Pueraria thunbergiana* flowers, used for diabetes, also contain tectoridin [60].

Aldose reductase (AR) is a key enzyme in the polyol pathway and plays an important role in diabetic complications. Tectoridin exhibits AR inhibitory activity. Jung et al. reported IC_50_ values of 1.08 × 10^−6^ M for tectoridin and 1.12 × 10^−6^ M for tectorigenin [61]. Moon et al. found that tectoridin-4′-O-β-D-glucoside had strong AR inhibitory activity (IC_50_ = 0.54 µM) [62].

These results indicate that tectoridin exerts antidiabetic activity through hypoglycemic, anti-hyperglycemic, and AR inhibitory effects. Furthermore, the aldose reductase inhibitory activity of tectoridin is achieved at very low concentrations; this concentration level remains pharmacologically active following oral administration, in stark contrast to other compounds that require high concentrations to exhibit pharmacological activity. However, a significant gap remains: there are no long-term studies evaluating whether treatment with tectoridin can reduce the incidence of clinically significant diabetic complications—such as cataract formation, neuropathy or nephropathy—or delay their progression in appropriate animal models. Furthermore, it remains unclear whether the hypoglycaemic effects reported for *Belamcanda chinensis* leaf extract are attributable to tectoridin itself or to other co-existing constituents [58]. Despite these limitations, aldose reductase inhibition remains a mechanism of action for tectoridin that offers both specificity and therapeutic potential, and should be prioritised in future preclinical development.

### 4.5. Antioxidant Activity

The Keap1/Nrf2 signaling pathway is a pivotal endogenous antioxidant defense mechanism. Tectoridin was identified as a novel Nrf2 agonist, significantly elevating Nrf2 activity (77.78 ± 4.80%) and enhancing antioxidant activity in CCl_4_-poisoned HepG2 cells [63]. Dong et al. found that tectoridin activated Nrf2 target genes and prevented lipid peroxidation [64]. Flavonoids are natural free radical scavengers, possibly due to their phenolic groups [65]. Niu et al. reported that fermented TCM by-products with high flavonoid content, including tectoridin, exhibited high total antioxidant activity [66]. Liu et al. also noted antioxidant activity in Astragalus sources rich in tectoridin [30]. Extracts from *Flos puerariae* and *Hovenia dulcis* containing tectoridin ameliorate oxidative stress by inhibiting MAPK pathway activation and restoring antioxidant enzyme activity [48]. Mykhailenko et al. identified tectoridin as an antioxidant in *Crocus sativus* leaf extract [23] and Ukrainian Iris species, which inhibited superoxide anion production and increased NRF2 expression [15]. Kim et al. isolated tectoridin (IC_50_ = 50.4 µM) as an antioxidant from *Maackia amurensis* [31]. *Belamcanda chinensis* root prevents biomolecule oxidation, and its active isoflavones, including tectoridin, showed high antioxidant activity in the ABTS assay and reduced serum transaminase activity and lipid peroxidation in rats [11]. Its capacity to directly activate the pivotal Keap1/Nrf2 defence pathway, combined with complementary free radical scavenging and enzyme regulatory activities, establishes a multi-faceted, potent protective mechanism against oxidative damage.

In a cell-free system, tectoridin exhibits moderate radical-scavenging activity (IC_50_ = 50.4 µM in the DPPH assay) [31], consistent with the general antioxidant behaviour commonly observed in many flavonoids. However, the pharmacological relevance of these direct antioxidant activities is limited by the high concentrations required, as well as the fact that such activities may be a characteristic shared by many polyphenolic compounds. Mechanistically compelling evidence identifies the Nrf2 signalling pathway as a key mediator of the antioxidant effects of tectoridin, although the specificity of Nrf2 activation by tectoridin and tectorigenin remains to be carefully defined. Future research should prioritise determining the precision of tectoridin’s interaction with Nrf2 activation and systematically evaluate the relationship between tissue concentrations achievable in relevant disease models and the pharmacological effects of Nrf2 pathway activation.

### 4.6. Effects on the Cardiovascular System

Cardiovascular diseases are a leading cause of death worldwide [67]. In vitro screening methods for anti-stroke drugs include lactate dehydrogenase (LDH) assays and PC12 cell models. Tectoridin is a potent LDH inhibitor and protects against PC12 cell injury [29]. Chen et al. showed that tectoridin increased PC12 cell survival and inhibited LDH release after oxygen-glucose deprivation/reoxygenation (OGD/R) (oxygen glucose deprivation, OGD), an effect attenuated by PI3K/AKT inhibitors. Tectoridin also increased Nrf2 levels, suggesting neuroprotection via PI3K/AKT signaling [68]. Li et al. reported that tectoridin from *Pueraria lobata* flowers increased the viability of OGD-induced PC12 cells from 31% to 67% [69]. Tang et al. concluded that *Belamcanda chinensis* extract and tectoridin are strong LDH inhibitors [29]. Tectoridin also inhibits MPP^+^-induced (mycoplasma pneumoniae pneumonia, MPP) cell death and apoptosis in PC12 cells [70].

Tectoridin from *Belamcanda chinensis* rhizomes has anti-angiogenic effects [11]. Jung et al. found that tectoridin and tectorigenin inhibited angiogenesis in chorioallantoic membrane and *Matrigel plug* assays, potentially by inhibiting endothelial cell proliferation [71]. Atherosclerosis is a major cause of cardiovascular disease [72].

Therefore, based on the above reports, tectoridin may be a candidate drug for the treatment of cardiovascular diseases. However, the translational significance of these observations is limited by two factors. Firstly, the effective concentrations observed in PC12 cells (10–50 µM) are unlikely to be achieved in brain tissue following oral administration of tectoridin [70], owing to its low systemic bioavailability and the potentially limited ability of its aglycone form to cross the blood–brain barrier. Second, no in vivo studies have yet evaluated the efficacy of tectoridin using models of ischaemic stroke, myocardial infarction or atherosclerosis. Although the anti-angiogenic findings are mechanistically intriguing, they have not yet been validated in relevant tumour angiogenesis models. Consequently, classifying tectoridin as a cardiovascular or neuroprotective agent remains speculative. Before any translational conclusions can be drawn, rigorous validation in clinically relevant in vivo models is essential.

### 4.7. Estrogenic Effects

Phytoestrogens are plant-derived compounds structurally similar to 17β-estradiol (E2) [73]. Tectoridin exerts estrogenic effects. Wang et al. demonstrated that tectoridin prevented estrogen-deficiency-associated bone loss [74]. Han et al. validated the anti-hot flush properties of tectoridin in an ovariectomy-induced mouse model via its estrogenic action [28]. Tectoridin can also modulate estrogen and thyroid receptors [32]. Shim et al. characterized the estrogenic activity of tectoridin in MCF-7 cells, showing that it activated reporter gene expression and decreased estrogen receptor protein levels [32]. Kang et al. found that tectoridin exerts estrogenic effects mainly through the rapid non-genomic estrogenic signaling pathway mediated by GPR30 and ERK, inducing potent effects in MCF-7 cells despite low binding to Erα [73]. Tectoridin is biotransformed into tectorigenin by intestinal flora, and this aglycone is then absorbed and exerts estrogenic effects [75]. These findings suggest tectoridin has potential to influence various diseases through estrogenic effects.

It is worth noting that tectoridin is considered a phytoestrogen capable of modulating oestrogen receptor-dependent signalling, with a mode of action that differs significantly from that of classical isoflavones. The above data indicate that tectoridin exerts its oestrogenic effects primarily via a rapid, non-genomic pathway dependent on GPR30, rather than through the classical ER-mediated genomic pathway. A key factor in the development of tectoridin is that the oestrogenic activity of tectoridin depends on its conversion by gut microbiota into tectorigenin [75]. This prodrug characteristic may offer therapeutic advantages by limiting unintended ER activation in extra-osseous tissues; however, it also introduces inter-individual variability driven by differences in gut microbiota composition, a factor that should be taken into account in the design of future clinical trials.

### 4.8. Other Pharmacological Effects

**Figure 4:**
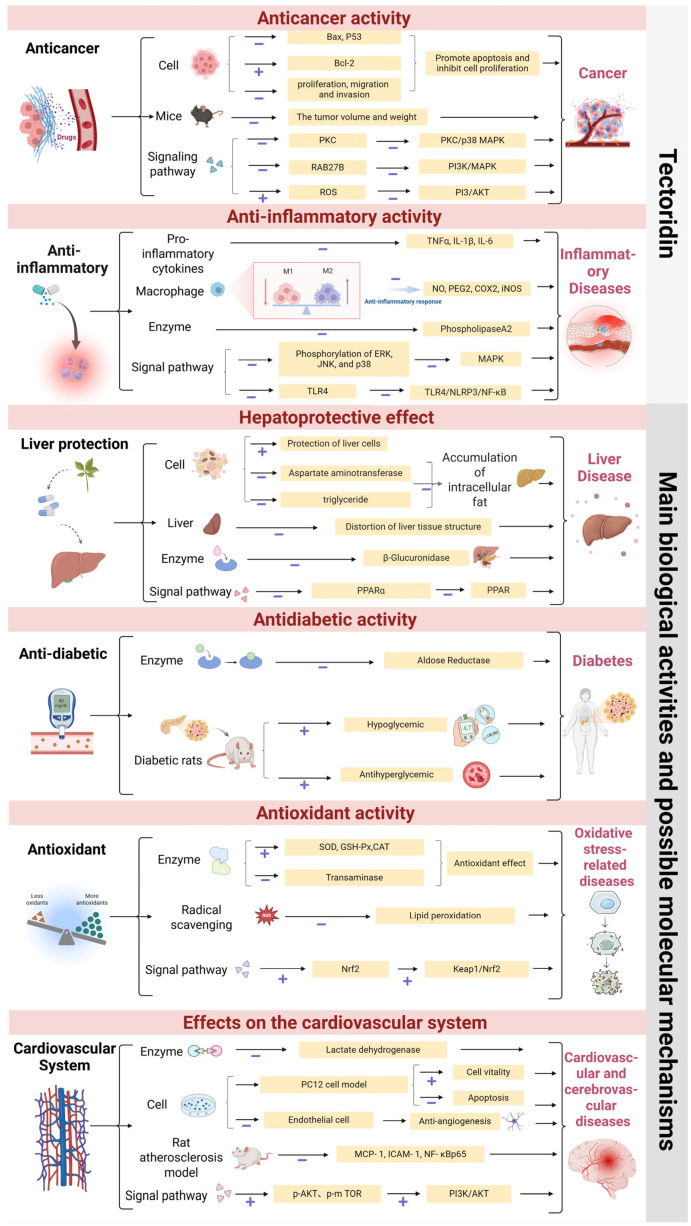
The main biological activities and possible molecular mechanisms of tectoridin. The molecular mechanisms and signalling pathways that may be involved in tectoridin, which has shown remarkable activities in anti-cancer, anti-inflammatory, antioxidant, anti-diabetic, hepatoprotective effects and neuroprotection, are presented, respectively. (“+”: promote, ”−“: inhibit).

Tectoridin has been investigated for other activities. Molecular docking suggests tectoridin may inhibit caspase-3 and induce apoptosis [33]. Dong et al. found tectoridin attenuated PM2.5-induced (Particulate Matter, PM) respiratory injury in mice, reducing IL-1β and TNF-α levels in bronchoalveolar lavage fluid and lung tissue damage [64]. Tectoridin exhibits defence activity against plant pathogenic microorganisms and functions as a biopesticide [76]. Mineral nutrient application can affect tectoridin biosynthesis in *Iris rhizomes* [77]. Tectoridin stimulates hair follicle cell activity and promotes hair shaft elongation [78]. It inhibits osteoclastogenesis and bone loss in ovariectomized mice [74]. Tectoridin shows antimutagenic [79] and antiplatelet activity [80]. It also exhibits anti-allergic effects, inhibiting hyaluronidase and passive cutaneous anaphylaxis [5,81]. It modulates skeletal and cardiac muscle sarcoplasmic reticulum calcium release channels [82]. These results indicate that tectoridin possesses diverse pharmacological activities worthy of further investigation. Key pharmacological effects are summarized in Figure 4.

### 4.9. Critical Evaluation of Target Specificity and Broad Pharmacological Effects

In addition to providing a comprehensive review of the pharmacological activities of tectoridin, it is necessary to further investigate whether the pharmacological effects of tectoridin and its aglycone, tectorigenin, result from genuine, target-specific regulation or are related to the non-specific, broad-spectrum pharmacological behaviour characteristic of many flavonoid compounds. Due to their polyphenolic structure, flavonoids are known to exhibit a range of physicochemical properties, including direct radical scavenging, metal chelation, electrophilic reactivity toward protein cysteine thiols, and perturbation of membrane lipid organization [83]. Given the wide-ranging biological activities of these flavonoids, it is essential that we establish a rigorous framework for evaluating the pharmacological activity of tectoridin.

Based on the integration of the aforementioned pharmacological activities, we have found that tectoridin exhibits specific pharmacological activity in the inhibition of aldose reductase (AR). Tectoridin and tectorigenin competitively inhibit aldose reductase in rat lenses with well-defined IC_50_ values (1.08 µM and 1.12 µM). Importantly, tectoridin-4′-O-β-D-glucoside exhibits markedly enhanced potency (IC_50_ = 0.54 µM), while structurally related irisolidone derivatives show minimal activity [61]. This suggests that AR inhibition is not a universal property of the isoflavone skeleton, but rather depends on specific structural features. Furthermore, in diabetic model rats, oral administration of tectoridin or tectorigenin significantly reduced the accumulation of sorbitol in the lens, sciatic nerves, and erythrocytes [61]. This suggests that tectoridin and its aglycone form possess specific molecular targets for inhibiting AR activity.

Conversely, the available evidence does not indicate that tectoridin has a specific role in regulating the MAPK and NF-κB signalling pathways. Numerous studies have reported that tectoridin treatment reduces the phosphorylation levels of components in the ERK, JNK, p38 and NF-κB pathways in various cellular and animal models of inflammation and cancer [4,45]. Although these observations are reproducible and pharmacologically significant, they do not in themselves constitute evidence of direct target binding. Reduced phosphorylation of a kinase in cellulo can arise from multiple indirect mechanisms: upstream receptor antagonism, modulation of phosphatase activity, altered cellular redox status, or even transcriptional downregulation of the kinase itself [84,85]. To date, we have not identified any published studies determining whether tectoridin binds directly to ERK2, JNK, p38, IKKβ or any other kinases in the MAPK/NF-κB cascade. Until such data are available, designating components of the MAPK or NF-κB pathways as direct targets of tectoridin remains speculative. Similarly, it is reasonable to propose that, regarding its antioxidant and Nrf2-activating properties, tectoridin’s ability to inhibit pro-inflammatory signalling may be mediated through redox mechanisms.

## 5. Pharmacokinetics of Tectoridin

### 5.1. The Pharmacokinetic Profile and Metabolism of Tectoridin

Pharmacokinetics plays a critical role in drug development. Liu et al. (2009) developed an HPLC method to determine tectoridin in rat plasma after intravenous injection [42]. For a dose of 25 µmol/kg, the peak concentration (C_max_) was 26.44 ± 5.67 µg/mL, half-life (T_1_/_2_) was 32.45 ± 9.06 min, and mean retention time (MRT) was 21.79 ± 3.21 min. AUC_0–t_, AUC_0–∞_, and C_max_ increased proportionally with dose, while MRT, T_1_/_2_, volume of distribution (V), and phase half-lives remained relatively stable [42]. Qu et al. investigated the pharmacokinetics of tectoridin and its metabolites after oral administration (200 mg/kg) in rats using HPLC/UV [43]. Tectoridin pharmacokinetics follows a first-order process with wide tissue distribution, rapid distribution, and relatively slow elimination.

Co-administration with other drugs can affect tectoridin’s pharmacokinetics. For example, co-administration with florfenicol resulted in lower plasma concentrations, reduced intestinal absorption, and increased clearance of florfenicol [86]. Yang et al. compared the pharmacokinetic profile of tectorigenin after oral administration of Iris tectorum Maxim. extract (ITME) versus pure tectoridin (PT). Plasma tectorigenin concentrations were significantly higher in the ITME group, along with increased half-life and AUC, indicating enhanced absorption due to co-existing components in the extract [14].

Tectoridin undergoes biotransformation to tectorigenin by intestinal bacteria or enzymes in the small intestine [53,87]. Chen et al. identified tectoridin metabolites in vivo and in vitro using LC-MS^n^. Urinary metabolites included tectorigenin, hydrogenated tectorigenin, hydroxylated tectorigenin, and their glucuronide/sulfate conjugates. Faecal metabolites included tectorigenin, di-hydroxylated tectorigenin, and sulfate-conjugated tectorigenin [88]. In addition, tectoridin can be metabolised into compounds such as tectorigenin-7-O-β-D-glucuronide (Te-7G) [89]. The major metabolic pathways and metabolites are listed in Table 2.

The aglycone tectorigenin often exhibits higher biological activity. For instance, oral tectoridin showed hepatoprotective effects, while intraperitoneal administration did not, likely due to conversion to tectorigenin in the gut [57]. Similarly, the aglycone form showed stronger anti-allergic activity [81]. Hydrolysis of the glycosidic moiety appears to enhance bioavailability and activity.

### 5.2. The Relationship Between the Pharmacokinetics and Pharmacological Activity of Tectoridin

The progress of tectoridin from a natural product with documented biological activity to a clinically viable therapeutic candidate hinges critically on the assessment of its pharmacokinetic limitations and their implications for the interpretation of its pharmacological activity.

First, a comprehensive review of the existing literature reveals a discrepancy between the concentrations required to produce direct pharmacological effects in vitro and those achievable in the systemic circulation following oral administration. Following oral administration of tectoridin (200 mg/kg) to rats, the peak plasma concentration of free tectorigenin glycoside was only approximately 8.7 µmol, and this occurred approximately 5 h after dosing [43]. The predominant circulating species are not the parent compound nor the free aglycone, but rather phase II conjugates, which achieve peak concentrations substantially higher than that of free tectorigenin. Excretion data further highlight the extensive metabolism of tectoridin: Within 72 h of oral administration (100–200 mg/kg), 14.2–14.7% of the dose was excreted in the urine in the form of 11 different metabolites [88]. Whilst this extensive Phase II metabolism facilitates elimination, it also raises key questions regarding the pharmacological activity of tectoridin itself; these questions remain largely unexplored. Crucially, as the primary mediator of tectoridin’s in vivo effects, tectorigenin has poor water solubility and low membrane permeability. These physicochemical characteristics explain why oral absorption remains limited even when the glycoside is administered directly in crystalline form. This underscores the need for advanced drug delivery strategies.

Second, in terms of anticancer, anti-inflammatory and antioxidant pharmacological activity, the effective concentration of tectoridin is typically in the range of 10–100 µM [4,37,40,41]. Following oral administration of tectoridin, peak concentrations in the systemic circulation reach approximately 2–3 µM, whilst steady-state concentrations are even lower [43]; consequently, the therapeutic concentration range in vivo is reduced by approximately one order of magnitude. This concentration gap has profound implications for the interpretation of the drug’s therapeutic potential. However, this discrepancy between pharmacokinetics and pharmacodynamics does not negate the therapeutic value of tectoridin; on the contrary, this characteristic makes it particularly suitable for the treatment of certain diseases. First, local effects within the gastrointestinal tract—the concentration of tectoridin in the intestinal lumen may far exceed systemic levels, which could yield therapeutic benefits in conditions such as inflammatory bowel disease or colorectal cancer without the need for systemic absorption. Second, transient yet pharmacologically significant concentrations of tectorigenin have been detected in the liver. This suggests that tectoridin possesses unique hepatoprotective activity, as evidenced by the consistent in vivo efficacy of orally administered tectoridin across multiple models of liver injury [54,56,57]. Conversely, challenges may arise in the treatment of metastatic cancers, neurodegenerative diseases requiring penetration of the blood–brain barrier, or chronic inflammatory diseases affecting extrahepatic tissues. In such cases, the inherent pharmacokinetic limitations of tectoridin must be overcome through formulation interventions or structural modifications.

## 6. Toxicity of Tectoridin

Extracts containing tectoridin from *Crocus sativus* leaves, *I. variegata*, and *I. hungarica* have shown cytotoxicity against triple-negative breast cancer (MDA-MB-231) and melanoma (IGR39) cell lines [15,23]. Tectoridin from *Clematis cirrhosa* ethanol extract exhibited cytotoxicity and pro-apoptotic ability against HT-29 colorectal cancer cells [33]. Liu et al. found tectoridin cytotoxic against PC3, MGC-803, Bcap-37, and MCF-7 cell lines [90]. Yang et al. reported that hot air drying (60 °C) of *Belamcanda chinensis* increased tectoridin content and reduced toxic side effects [91]. Pan et al. reported no cytotoxic or pro-apoptotic effects on Raw 264.7 cells even at 200 µM, suggesting safety in medicinal foods [51]. Lee et al. showed that tectorigenin exhibited cytotoxicity, while tectoridin did not, indicating the aglycone structure is essential for cytotoxic properties [92]. Overall, while cytotoxic to some cancer cells, tectoridin appears to have low general toxicity, particularly in dietary or moderate medicinal use. However, more rigorous in vivo toxicity studies are needed.

## 7. Strategic Recommendations for Advancing the Clinical Translation of Tectoridin

Natural products are of great importance to human health and wellbeing [93]. Advancing the transition of tectoridin from nature to clinical application will provide fresh perspectives for the development and utilization of natural medicines. Tectoridin, as one of the primary active constituents in the *Belamcanda chinensis* and *Pueraria lobata*, exhibits multi-target pharmacological activity, establishing a robust scientific foundation for its translational development [94]. However, its inherent pharmaceutical limitations constitute the primary obstacle. *Oral tectoridin* exhibits extremely low bioavailability, as its glycoside form necessitates hydrolysis by gut microbiota into the active aglycone tectorigenin prior to absorption [95]. Therefore, its clinical translation requires systematic design and collaborative research efforts.

First, at the molecular level, structural chemical modification is a fundamental strategy. Dong et al. have successfully synthesized tectoridin and its derivatives through total synthetic approaches [3], enabling subsequent structure-activity relationship studies and structural optimization. Research indicates that modifying tectoridin through sulfonation significantly enhances its water solubility and in vitro antioxidant activity, pointing toward improved physicochemical properties [5]. Second, at the formulation level, developing advanced drug delivery systems represents a highly promising avenue [96]. Given that tectoridin undergoes microbial transformation in the colon [95], designing colon-targeted delivery systems (such as pH-dependent or microbiota-triggered formulations) could protect the parent drug until it reaches the site of action, enhancing conversion efficiency and local efficacy [97]. Furthermore, Zhu et al. indicate that tectorigenin can treat metabolic-associated steatohepatitis by modulating the hepatic-enteric axis [98]. Pharmacokinetic studies further confirmed that its metabolites are excreted via bile and undergo enterohepatic circulation [99]. These findings provide a rationale for designing liver-targeted drug delivery systems to enhance therapeutic efficacy in liver diseases.

Pharmacokinetic studies by Qu et al. revealed that following oral administration of tectoridin, the blood concentrations of its metabolites (such as tectorigenin) were significantly higher than those of the parent drug [43], confirming its prodrug properties. As previously mentioned, conversion to tectorigenin enhances biological activities such as antioxidant properties, hepatoprotection, and anti-allergic effects [5,57,81]. Therefore, focusing on the conversion of tectoridin to its aglycone form will facilitate its clinical translation. Liu et al. found that *L. reuteri* DSM20016, *L. rhamnosus* GGB41031 and *B. adolescentis* ATCC15703 have great potential for converting tectoridin from *Pueraria flos* to the more bioactive tectorigenin [95]. This suggests that future formulation development must not only focus on prodrug delivery but also consider optimizing its conversion into the active end product and its distribution.

Furthermore, ensuring the sustainability and quality control of raw materials is crucial. This includes employing modern synthetic biology strategies for large-scale production and establishing comprehensive quality control standards from plant-based raw materials to finished pharmaceutical preparations [100].

## 8. Analysis of the Mechanisms Regulated by Tectoridin

**Figure 5:**
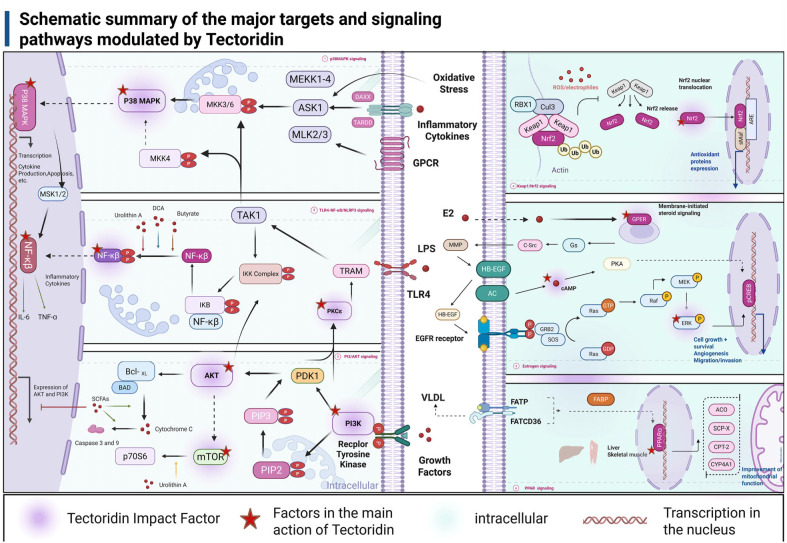
Schematic summary of the major targets and signaling pathways modulated by tectoridin. The molecular mechanism and main targets of tectoridin in regulating MAPK, NFκB, PI3K/AKT, Keap1/Nrf2, ERK, and PPARɑ signalling pathways are demonstrated in detail, and the associations between the pathways regulated by tectoridin were revealed.

As mentioned earlier, the section on pharmacological activity has systematically outlined the various signalling pathways involved in the pharmacological effects of tectoridin. Figure 5 summarizes proposed molecular mechanisms, providing a theoretical foundation for future research.

However, when these pathways are presented as independent and parallel, they remain mechanistically fragmented and fail to address a fundamental question: do these effects stem from specific molecular interactions with particular protein targets, or do they reflect indirect cellular responses propagated through interconnected signalling networks? This paper synthesises the existing evidence into a coherent, hierarchical framework designed to distinguish between outcomes arising from validated direct targets, convergent signalling nodes, and signalling networks.

### 8.1. Nrf2/Keap1 Signalling Pathway

A growing body of evidence suggests that the Keap1/Nrf2 pathway is not merely one of the pathways responsive to tectoridin, but is more likely to be a central hub linking antioxidant and anti-inflammatory effects. In the aforementioned analysis, tectoridin was shown to activate Nrf2 target genes and to protect against PM2.5-induced ferroptosis both in vitro and in vivo. A more compelling piece of evidence is that these protective effects were almost entirely lost in Nrf2 knockout mice or in cells treated with Nrf2 siRNA [64,101]. Furthermore, Nrf2 activation is not an isolated event; there is an interaction between it and the NF-κB signalling pathway, a relationship that may explain the frequently observed co-regulation of inflammatory and oxidative stress endpoints in studies. Electrophilic Nrf2 inducers can suppress NF-κB transcriptional activity through multiple mechanisms, including the Nrf2-dependent induction of heme oxygenase-1 (HO-1), which generates anti-inflammatory carbon monoxide and biliverdin, as well as direct competition for transcriptional coactivators such as p300/CBP [102,103,104]. Conversely, NF-κB can also downregulate Nrf2 signalling by promoting the nuclear translocation of Keap1 or by competing for binding to antioxidant response elements [104,105]. This two-way dialogue suggests that the anti-inflammatory effects of tectoridin may stem from a unified redox regulatory mechanism centred on Nrf2 activation.

### 8.2. ERK Signalling Pathway

The ERK signalling pathway occupies a key position at the intersection of multiple response pathways for tectoridin. As described above, in a mouse model of collagen-induced arthritis, treatment with tectoridin significantly reduced paw and joint swelling and alleviated tissue damage; these effects were associated with the inhibition of ERK, JNK and p38 phosphorylation. Similarly, in studies of inflammation-related tissue damage, tectoridin has been shown to inhibit the phosphorylation of MAPK components, a finding associated with its overall anti-inflammatory activity [4]. This suggests that the activation of ERK is associated with inflammatory signalling.

However, designating ERK as a direct target of tectoridin remains uncertain, owing to the lack of direct binding data and the compound’s distinct pharmacokinetic behaviour. Tectoridin has poor oral bioavailability; following oral administration, the parent compound is almost entirely converted to its aglycone form, tectorigenin, in the systemic circulation. Consequently, any ERK phosphorylation modulation observed in vivo following oral administration of tectoridin is more reasonably attributed to tectorigenin or its conjugated metabolites, rather than to tectoridin itself. Until direct binding evidence is obtained for either tectoridin, tectorigenin or downstream metabolites, regulation of the ERK pathway should be regarded as a pharmacodynamic readout rather than evidence of direct target binding.

### 8.3. AR and Calcium-Release Channels

In stark contrast to the aforementioned regulatory mechanisms at the pathway level, only a small number of molecular targets have been validated through direct binding assays. Among these, aldose reductase has been identified as a direct target, with its competitive inhibition confirmed by well-defined IC_50_ values and structure-activity relationship data [61]. Furthermore, tectoridin directly modulates the calcium release channels (rhanodine receptors, RyR) in skeletal and cardiac muscle. The IC_50_ value of tectoridin for skeletal muscle RyR1 is 17.3 ± 1.3 µM (Kd = 6. 7 ± 0.4 µM), whilst its affinity for cardiac RyR2 is approximately three times higher (IC_50_ = 5.2 ± 0.6 µM; Kd = 0.95 ± 0.3 µM) [82]. This is the only direct binding interaction, apart from AR, for which affinity constants have been clearly characterised to date. It is worth noting that tectorigenin binds more tightly to HSA than tectoridin, providing biophysical evidence for the differences in protein recognition between the glycoside and its aglycone, which may explain the differences in their pharmacokinetic and pharmacodynamic profiles [106].

Based on the above evidence, this paper systematically integrates the framework of tectoridin mechanisms. First, it proposes the Keap1/Nrf2 pathway as the integrative hub for redox signalling. Second, it identifies direct molecular targets validated by binding affinity data: aldose reductase and the ryanodine receptor. Third, it encompasses downstream network effects, including the regulation of NF-κB transcriptional activity, alterations in MAPK phosphorylation, and changes in PI3K/AKT signalling. This explains why a single molecular entity can exhibit seemingly diverse pharmacological activities across different disease models without the needing to bind to multiple targets, and fills a knowledge gap regarding the mechanism of action of tectoridin. This integrated analysis is crucial for both the credibility of the mechanism and the rational development of tectoridin -based therapeutics.

## 9. Conclusions and Future Perspectives

Drug discovery from natural products represents a significant source of new therapeutics, recognized for their reliable effects and favourable safety profiles [107]. Tectoridin is an important natural compound isolated from herbs such as *Belamcanda chinensis* (L.) DC, *Pueraria lobata* (Willd.) Ohwi, and *Iris tectorum* Maxim [10,12,26]. This review comprehensively summarizes its chemical properties, botanical sources, pharmacological activities, toxicity, and pharmacokinetics. Studies indicate diverse effects, including anticancer, anti-inflammatory, hepatoprotective, antidiabetic, antioxidant, cardiovascular, and estrogenic activities.

Cancer remains a leading cause of death worldwide [108]. Tectoridin demonstrates promising anticancer effects by regulating apoptosis-related proteins, modulating signaling pathways, and inhibiting cancer cell proliferation, migration, and invasion. Chronic inflammation is a key driver of cancer development [109]. The significant anti-inflammatory activity of tectoridin supports its potential as an anticancer agent. However, current studies on its anticancer effects are limited to cellular levels. Further in vivo animal studies are needed to elucidate its molecular mechanisms. Although promising, no clinical trials have confirmed its anticancer efficacy in humans. Future research should establish its dose–effect relationship to identify optimal dosing and facilitate clinical translation.

The therapeutic efficacy of tectoridin against inflammatory diseases has been established through extensive experimental models. It significantly reduces pro-inflammatory factors such as IL-6, TNF-α, and IL-1β, inhibits M1 macrophage polarization, and suppresses the NF-κB pathway. However, current clinical applications primarily involve oral administration of tectoridin-containing herbs rather than the purified compound. Further clinical investigations are warranted to evaluate the therapeutic potential of tectoridin alone.

Despite robust preclinical evidence, current research on tectoridin remains largely confined to cell cultures and animal models. Moreover, relatively few in vivo studies of this activity have been conducted. Only a handful of studies have progressed to preclinical or clinical pharmacodynamic evaluations, underscoring an urgent need for further research into the clinical application. Furthermore, another point worth noting is that although a substantial body of preclinical literature documents its pharmacological activity in various cellular and animal models, a systematic search of databases such as PubMed and Web of Science conducted for this review failed to identify any reports of clinical studies on tectoridin itself. Nevertheless, clinical literature on herbal preparations containing tectoridin provides insightful, albeit indirect, evidence. A study of Shegan Liyan oral liquid (derived from *Belamcanda chinensis*) involving pediatric patients with acute pharyngitis reported an efficacy rate of approximately 83% in the treatment group [110]. Similarly, a meta-analysis of Shegan Mahuang decoction—a classic traditional Chinese medicine formula containing *Belamcanda chinensis*—has further demonstrated improved efficacy and reduced adverse events compared with conventional therapy in pediatric cough-variant asthma [111]. Although these studies suggest that herbal preparations containing tectoridin may offer clinical benefits, they cannot be regarded as direct evidence of the therapeutic efficacy of tectoridin itself. Therefore, the pharmacological activity summarised in this review should be defined as potential therapeutic promise rather than validated indications for human use. Future research should prioritise rigorous clinical trials to evaluate the safety, tolerability and pharmacokinetics of oral tectoridin in healthy volunteers.

Advancing tectoridin to clinical use requires establishing a systematic translational pathway. The logical starting point lies in defining its clinical positioning: focusing on specific indications where mechanisms are relatively well-defined and clinical needs are urgent, such as ischaemic stroke or metabolic inflammation. However, tectoridin’s low oral bioavailability and reliance on gut microbiota hydrolysis result in uncertainty regarding therapeutic efficacy and significant inter-individual variability. The core challenge lies in overcoming its inherent druggability limitations through three primary approaches: first, structural modification; second, developing advanced drug delivery systems; and thirdly, exploring co-administration strategies to influence its pharmacokinetics.

Beyond molecular and formulation strategies, advancing the industrialization of natural medicines requires ensuring the sustainability of raw materials and quality controllability. Established total chemical syntheses can provide high-purity, stable starting materials. Alternatively, resource recovery strategies, such as reclaiming materials from medicinal herb residues, represent viable approaches. Moreover, as a key active component in traditional herbs, tectoridin may be explored for development into quality-controlled modern Chinese medicinal preparations, representing a distinctive and practical translation pathway.

Drug safety is essential for therapeutic development. Although natural compounds like tectoridin are generally associated with fewer toxic side effects, the potential toxicity of isoflavone glycosides warrants attention. Currently, no significant toxicity has been reported in medicinal uses, yet tectoridin exhibits cytotoxicity in various cell lines. Most toxicity assessments remain at the cellular level, underscoring the need for rigorous in vivo studies.

In conclusion, tectoridin possesses diverse biological activities. Data from in vitro and in vivo experiments indicate considerable therapeutic potential for a wide range of human diseases. To advance its clinical development, future research should focus on: (1) elucidating molecular mechanisms through in vivo experiments for prioritized indications like cancer, and strengthening dose–response relationship studies; (2) conducting clinical trials to evaluate its efficacy in inflammatory diseases; (3) performing rigorous in vivo toxicity studies to confirm safety; and (4) promoting its effective transition through chemical modification, drug delivery system development, and combination therapies.

## Figures and Tables

**Table 1:** The main plant source of tectoridin.

Plant Species	Family	Used Part	Extract	Refs.
*Belamcanda chinensis* (L.) Redouté	Iridaceae	Rhizome and leaves	70% ethanol	[10]
*Iris tectorum* Maxim	Iridaceae	Rhizome and root	70% ethanol	[10,12,14]
*Iris pallida* Salisb	Iridaceae	Rhizome	10 mL methanol	[15]
*Iris hungarica* Waldst. & Kit	Iridaceae	Rhizome	10 mL methanol	[15]
*Iris sibirica* L.	Iridaceae	Rhizome	10 mL methanol	[15]
*Iris variegata* L.	Iridaceae	Rhizome	10 mL methanol	[15]
*Iris confusa* Sealy	Iridaceae	Underground parts	75 mL methyl tert-butyl ether and 25 mL methanol (3:1, vol/vol), 75 mL water and 25 mL methanol (3:1, vol/vol).	[8]
*Iris hybrida* Retz	Iridaceae	Leaves	Methanol	[16]
*Iris × germanica* L.	Iridaceae	Rhizome	95% ethanol	[17]
*Iris spuria* L.	Iridaceae	Rhizomes	40–60 °C petroleum ether, CHC_I3_ 70%aq. MeOH	[18]
*Iris pseudacorus* L.	Iridaceae	Leaves	96% ethanol	[19]
*Iris crocea* Jacquem. ex R.C.Foster	Iridaceae	Rhizomes	Methanol (4 × 5 L)	[20]
*Iris humilis* Georgi	Iridaceae	Rhizomes, above-ground Vegetative parts (stem and leaf) and flowers	80% MeOH, 80% acetone, 52% perchloric acid, distilled water	[21]
*Iris pumila* L.	Iridaceae	Rhizomes, above-ground Vegetative parts (stem and leaf) and flowers	80% MeOH, 80% acetone, 52% perchloric acid, distilled water	[21]
*Iris albicans* Lange	Iridaceae	Leaves	70% ethanol	[22]
*Crocus sativus* L.	Iridaceae	Leaves	ethanol/water 70/30 (*v/v*)	[23]
*Viola mandshurica* W.Becker	Violaceae	The whole plant	Three volumes of distilled water and n-hexane (12 g), CHCl_3_ (1.5 g), EtOAc (8.7 g), BuOH (34 g)	[24]
*Pueraria montana* (Lour.) Merr	Fabaceae	Flowers	70% methanol	[25]
*Pueraria lobata* (Willd.) Ohwi	Fabaceae	Dried flowers	Ethanol	[6,7,26]
*Pueraria lobate* (Willd.) Ohwi	Fabaceae	Dried flowers	70% ethanol, 50% methanol	[27]
*Pueraria thomsonii* Benth	Fabaceae	Dried flowers	70% ethanol, 50% methanol	[27]
*Puerariae Lobatae* Radix	Fabaceae	Dried root	70% ethanol	[29]
*Astragalus membranaceus* Fisch. ex Bunge	Fabaceae	Rhizoma	60% ethanol	[29]
*Astragalus membranaceus var. mongholicus* (Bunge) P.K.Hsiao	Fabaceae	Flower	70% methanol	[30]
*Maackia amurensis* Rupr	Fabaceae	Dried bark	70% ethanol	[31]
*Glycine max* (L.) Merr	Fabaceae	Plant aerial parts	70% ethanol	[33]
*Clematis cirrhosa* L.	Ranunculaceae Juss	Plant aerial parts	70% ethanol	[33]
*Scutellaria baicalensis* Georgi	Labiatae	Roots and leaves	Methanol:water:formic acid (70:29:1)	[34]
*Vaccinium bracteatum* Thunb	Ericaceae Juss	Leaves	95% ethanol	[35]
*Psammosilene tunicoides* W. C. Wu et C. Y. Wu	Caryophyllaceae	Dried aerial parts	80% ethanol	[36]

**Table 2:** The major metabolic pathways and metabolites of tectoridin.

Intake Pattern	Animal Model	Dose	Metabolic Pathway	Main Metabolites	Ref.
gavage	Male Sprague–Dawley rats	100 mg/kg	urine	tectorigenin-7-O-β-Dglucuronide (Te-7G)and tectorigenin	[88]
gavage	Male Sprague–Dawley rats	200 mg/kg	urine	tectorigenin-7-O-β-Dglucuronide (Te-7G), tectorigenin and 6-OH Biochanin A-glucuronide (6-OH BiA-G)	[89]
gavage	Male Sprague–Dawley rats	100 mg/kg	bile	tectorigenin-7-O-β-Dglucuronide (Te-7G)	[89]
gavage	Male Sprague–Dawley rats	200 mg/kg	bile	tectorigenin-7-O-β-Dglucuronide (Te-7G)	[89]
gavage	Male Sprague–Dawley rats	200 mg/kg	bile	tectorigenin-7-O-glucuronide-4′-O-sulfate (Te-7G-4′S)	[42]
gavage	Male Sprague–Dawley rats	200 mg/kg	blood	tectorigenin-7-O-β-Dglucuronide (Te-7G), tectorigenin-7-O-sulfate (Te-7S), and tectorigenin	[42]
oral administration	male Sprague-Dawley (SD) rats	32 mg/kg	plasma	tectorigenin-7-O-glucuronide-4′-O-sulfate (Te-7G-4′S), tectorigenin-7-O-β-Dglucuronide (Te-7G), tectorigenin-7-O-sulfate (Te-7S) and tectorigenin	[13]
gavage	Wistar rats (male and female)	100 mg/kg	faeces	tectorigenin	[87]
gavage	Wistar rats (male and female)	100 mg/kg	urine	tectorigenin, di-hydroxylated tectorigenin and sulfate-conjugated tectorigenin	[87]
orally administered	Male Sprague–Dawley (SD) rats	40 mg/kg	urine	tectorigenin, hydrogenated tectorigenin, mono-hydroxylated tectorigenin, di-hydroxylated tectorigenin, glucuronide-conjugated tectorigenin and sulfate-conjugated tectorigenin	[80]
orally administered	Male Sprague–Dawley (SD) rats	250 mg/kg	urine	tectorigenin	[80]

## Data Availability

No new data were created or analyzed in this study.
